# The effect of incomplete blinking rate on corneal epithelial remodeling after trans-PRK surgery: a six-month study

**DOI:** 10.3389/fmed.2023.1305461

**Published:** 2024-01-08

**Authors:** Fan Yang, Huanmin Cheng, Shaozhen Zhao, Yue Huang

**Affiliations:** Tianjin Key Laboratory of Retinal Functions and Diseases, Tianjin Branch of National Clinical Research Center for Ocular Disease, Eye Institute and School of Optometry, Tianjin Medical University Eye Hospital, Tianjin, China

**Keywords:** Trans-PRK, corneal epithelial thickness, incomplete blinking rate, blinking times, lipid layer

## Abstract

**Purpose:**

To evaluate the impact of incomplete blink rate on corneal epithelial thickness after transepithelial photorefractive keratectomy (Trans-PRK) surgery.

**Methods:**

Trans-PRK patients were divided into two groups based on preoperative incomplete blinking rates, namely rates ≤0.5 (41 right eyes, group A) and rates >0.5 (65 right eyes, group B). We used anterior segment optical coherence tomography to measure the corneal epithelial thickness (CET) and lipiview to measure the number of blinks, incomplete blinking rate, and lipid layer thickness (LLT).

**Results:**

In both groups, CET decreased at 1 week and 1 month, and the thicknesses in the IT, T, and ST regions exceeded the preoperative levels. Three months after Trans-PRK, the thickness exceeded the preoperative levels and continued to increase during subsequent follow-ups. The blinking times and LLT in both groups decreased at 1 week and gradually increased but did not return to preoperative levels. Group A maintained an incomplete blinking rate of over 0.5 at all postoperative periods. The incomplete blinking rate of group B remained above 0.5 postoperatively, although there was an improvement compared with preoperative rates. Both groups showed a correlation between changes in CET and incomplete blinking rate. There was a correlation between changes in LLT and the incomplete blinking rate after surgery.

**Conclusion:**

Both groups showed uneven corneal epithelia thickening, which became more pronounced approaching the peripheral areas. In addition, changes in CET after surgery were positively correlated with the incomplete blinking rate in both patient groups. There was a negative correlation between postoperative LLT and incomplete blinking rate.

## 1 Introduction

Refractive laser surgery, including transepithelial photorefractive keratectomy (Trans-PRK), femtosecond laser-assisted *in situ* keratomileusis (FS-LASIK), and small incision lens extraction (SMILE), has become the primary method for correcting refractive errors. Compared to Trans-PRK, FS-LASIK, and SMILE have advantages such as milder pain and quicker postoperative visual recovery. However, Trans-PRK remains the primary surgical method for patients with thinner corneas. In Trans-PRK one-step epithelial ablation, the postoperative corneal epithelium compensates for the irregularity of the stromal surface shape by changing its thickness, causing changes in the corneal epithelial thickness (CET) (1).

Blinking is a coordinated movement of closing and opening the eyelids in a natural state and is divided into two types: voluntary blinking and reflexive closing movements. Spontaneous blinking can be classified as complete or incomplete. Normally, adults blink 15–20 times per minute. When the blink rate or amplitude exceeds or falls below the normal value, it is considered abnormal. According to reports, the integrity of the cornea or the condition of the tear film on the surface of the cornea and conjunctiva can affect the blinking rate (2). Blinking maintains the integrity of the eye surface, helps to maintain eye surface humidity, facilitates tear drainage, and regulates the expression and distribution of meibomian gland lipids (3). The lipid layer of the tear film spreads across the ocular surface, with each blink delaying the evaporation of tears and improving the stability of the tear film (4–6). Trans-PRK removes the epithelium and damages the corneal nerves. The decrease in corneal sensitivity can affect blinking times and incomplete blinking rate, which is an important factor leading to instability of the tear film. Additionally, incomplete blinking may lead to defects in the redistribution of mucin and lipid layers, increasing tear evaporation (7). The change in tear film stability caused by nerve damage can lead to ocular surface inflammation, and the subsequent increase in epithelial cell renewal may lead to corneal epithelial proliferation and thickening.

The interaction between the eyeball surface and eyelids is important for maintaining tear production and flow (8). Corneal refractive surgery can cause corneal flattening or morphological changes, leading to uncoordinated interactions between the ocular surface and eyelids (3, 9, 10). Some scholars have proposed that mechanical friction caused blinking rate increase can cause CET; however, currently, there is no research focusing on how changes in the blinking pattern of patients after refractive surgery affect CET. Therefore this study assess the changes in the postoperative incomplete blinking rate in both groups and the impact on postoperative lipid layer and epithelial thickness.

## 2 Materials and methods

This prospective studyrecruited and enrolled 106 consecutive xpatients visiting Tianjin Medical University Eye Hospital between March 2022 and April 2023, and these patient independently chose Trans-PRK as the treatment method. Therefore, we divided patients who underwent Trans-PRK surgery into two groups based on whether the preoperative incomplete blinking rate was >0.5. Patients were divided into two groups: 41 patients (41 right eyes) with a preoperative incomplete blinking rate ≤ 0.5 formed group A, and 65 patients (65 right eyes) with a preoperative incomplete blinking rate > 0.5 formed group B. All patients underwent preoperative and postoperative examinations, including uncorrected vision, best-corrected vision, dominant and cycloplegic diopters, non-contact intraocular pressure, and corneal topography using the Scheimpflug tomography system (Pentacam; Oculus GmbH, Wetzlar, Germany).

Each patient provided written informed consent. The clinical examinations performed in this study complied with the Declaration of Helsinki and were approved by the Ethics Committee of the Eye Hospital of Tianjin Medical University (2022KY-09).

### 2.1 Inclusion–exclusion criteria

Patients with low to moderate myopia (spherical diopter −0.50D to −6.00D, columnar diopter 0.00D to −2.00D) aged between 18 and 45 years were included in this study. Additional requirements included patients having undergone no previous ophthalmic surgery, maintained refractive stability for at least 2 years, and discontinued contact lenses for at least 4 weeks. Patients with abnormal or keratoconus topography, active eye inflammation, and systemic diseases that may affect corneal wound healing were not eligible for surgery.

### 2.2 Surgical procedures

All surgeries were performed by a professional ophthalmologist. Preoperative gatifloxacin eye drops (China OTSUKA Pharmaceutical Co., Ltd.) were administered four times daily for three consecutive days and diquafosole sodium eye drops (Santen Pharmaceutical Co., Ltd.) four times a day for 3 days (depending on the patient’s dry eye symptoms). Intraoperative anesthesia was administered using 0.4% bupivacaine hydrochloride eye drops. All operations were performed using the standard aspheric aberration-free mode of Trans-PRK, and Smart Pulse Technology (SPT) was used for laser ablation of the epithelium and stroma using an Amaris 1050 excimer laser (). After laser ablation, the eye surface was thoroughly cleaned with a cooled equilibrium salt solution, bandaged contact lenses were applied (PureVision, Bausch & Lomb) and 0.3% tobramycin-dexamethasone eye drops were administered. The Bandaged contact lenses were removed on the third day after surgery. All patients received a local infusion of levofloxacin for 7 days, 0.1% fluromethalone four times a month (then reduced once a month until the fourth month), and 0.3% sodium hyaluronate eye drops for 4 months.

### 2.3 Optical coherence tomography

The RTVue optical coherence tomography (OCT) (Optovue Inc., Fremont, CA, United States), which is based on the principle of low-coherence light, can provide a cross-sectional image of the cornea and measure the thickness of the epithelium using its built-in measurement tool. The epithelial thickness map was generated using an automatic algorithm and divided into 17 regions: a central region with a diameter of 2 mm, eight regions evenly distributed within the area between the 2 and 5 mm diameter rings, and eight regions evenly distributed within the area between the 5 and 6 mm diameter rings, including, S (superior), N (nasal), T (temporal), I (inferior), SN (superonasal), IN (inferotemporal), IT (inferonasal), and ST (superotemporal).

### 2.4 Incomplete blinking rate and lipid layer thickness

All patients were examined by a professional operator. A LipiView II ocular surface interferometer (TearScience Inc., Morrisville, NC, United States) was used to measure lipid layer, incomplete blinking rate and blinking times. For 20 s, the maximum, minimum, and average values of lipid layer thickness (LLT), and the number of incomplete and total blinks were measured. Normally, adults blink 15–20 times per minute. The correct blinking action is that the upper eyelid should touch the lower eyelid, and the eyelid should be closed. If the upper and lower eyelids are not completely closed, it is called incomplete blinking. Abnormal blinking was defined as incomplete blink rate > 0. The majority of patients with an incomplete blinking rate > 0 after refractive surgery were divided into two groups according to the degree of incomplete blink: an incomplete blink rate lower than 0.5 and an incomplete blink rate higher than 0.5.

### 2.5 Meiboscore

The degree of meibomian gland loss was measured using an Oculus Keratograph 5 M topographer (Oculus Optikgeräte GmbH). Partial or complete loss of the meibomian glands for each eyelid was scored using the following grades (meiboscore): level 0, no glandular atrophy; level 1, ≤25% glandular atrophy; level 2, 26 to 50% glandular atrophy; level 3.51 to 75% glandular atrophy; and level 4, ≥ 75% glandular atrophy.

## 3 Statistical analysis

All statistical analyses were performed using SPSS version 19 (IBM Corp., Armonk, NY, United States). The normality of the data was verified using the Kolmogorov–Smirnov test. Two-sample *t*-tests with unequal variance were applied for normally distributed data. Nonparametric tests were applied for non-normally distributed data. A repeated-measures two-way analysis of variance (ANOVA) was used to analyze the overall variation of the parameters, and Pearson correlation coefficients were used to analyze the linear relationships between the deformation parameters. In the correlation analysis, both eyes would lead to significantly correlated results. On the premise that the sample size was large enough, we chose a monocular study to avoid this error. Statistical significance was set at *p* < 0.05.

## 4 Results

**Figure 1:**
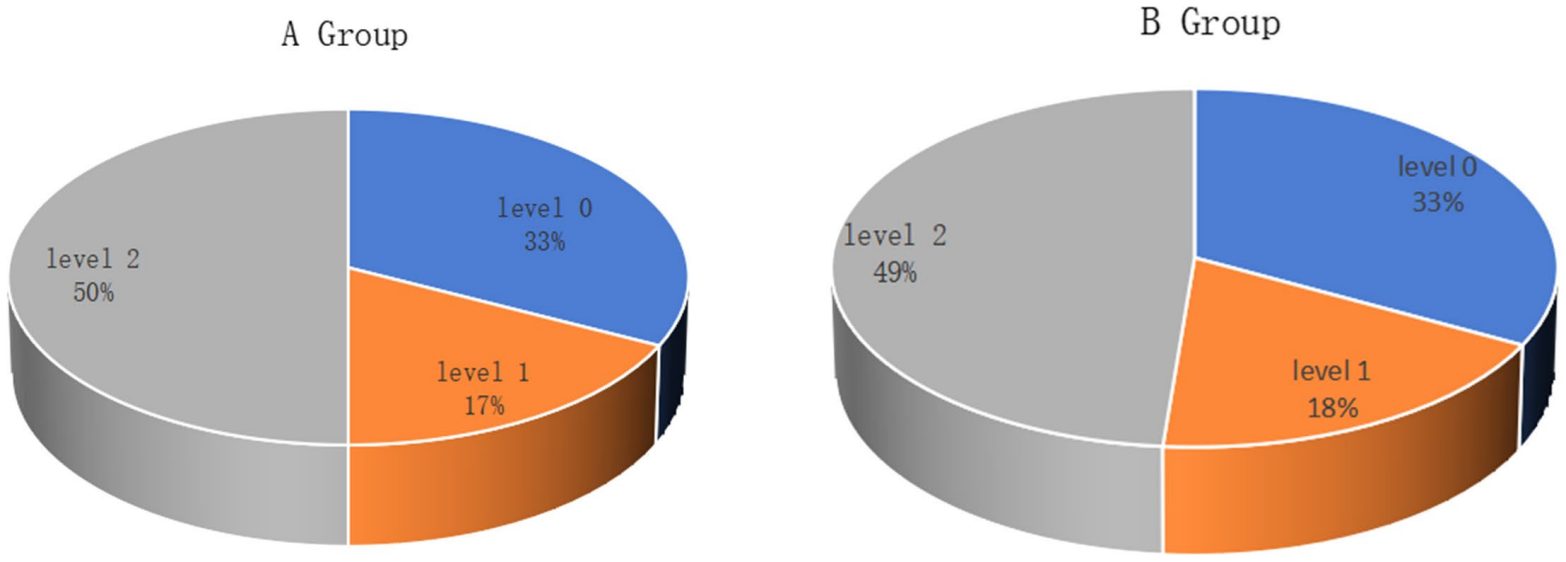
Degree of meibomian gland loss in both groups.

A total of 106 patients (106 right eyes) underwent Trans-PRK to correct low-to-moderate myopia and were divided into two groups A and B according to incomplete blinking rates. There were no differences in the LLT and the degree of meibomian gland loss between the two groups (Figure 1). Table 1 presents baseline characteristics including age, spherical equivalent (SE), astigmatism, central corneal thickness (CCT), central epithelial thickness (CET), and uncorrected distance visual acuity (UDVA). There were no significant differences in baseline characteristics between the groups.

**Table 1:** Demographic information and research parameters of patients with myopia receiving Trans-PRK treatment.

	Incomplete blinking rate ≤ 0.5	Incomplete blinking rate > 0.5	*F*	*p*	95%CI
Number of eyes	41	65			
Age (years)	27.95 ± 6.58	26.75 ± 7.04	0.58	0.45	−1.48 to 3.88
Sex (F:M)	20:21	37:28	1.05	0.41	–
SE (D)	−2.96 ± 1.47	−3.36 ± 1.17	2.46	0.12	−3.46 to −2.95
Astigmatism (D)	−0.93 ± 0.50	−0.80 ± 0.48	0.19	0.67	−0.35 to 0.08
CCT (μm)	526.54 ± 31.38	522.49 ± 40.17	1.91	0.17	−9.82 to 17.91
CET (μm)	53.24 ± 2.83	51.87 ± 0.24	3.72	0.06	0.38 to 2.38
UDVA (logMAR)	0.20 ± 0.14	0.16 ± 0.02	0.28	0.60	−0.02 to 0.10
Axial length (mm)	24.77 ± 0.99	25.04 ± 0.87	0.31	0.86	−0.63 to 0.11
Pupil diameter (mm)	6.10 ± 0.84	6.61 ± 1.85	0.20	0.66	−1.01 to −0.02
Lipid layer thickness (mm)	75.32 ± 21.75	69.62 ± 20.34	0.58	0.45	−2.51 to 13.90

Statistical significance was set at *p* < 0.05.

### 4.1 Differences in corneal epithelial thickness

**Figure 2:**
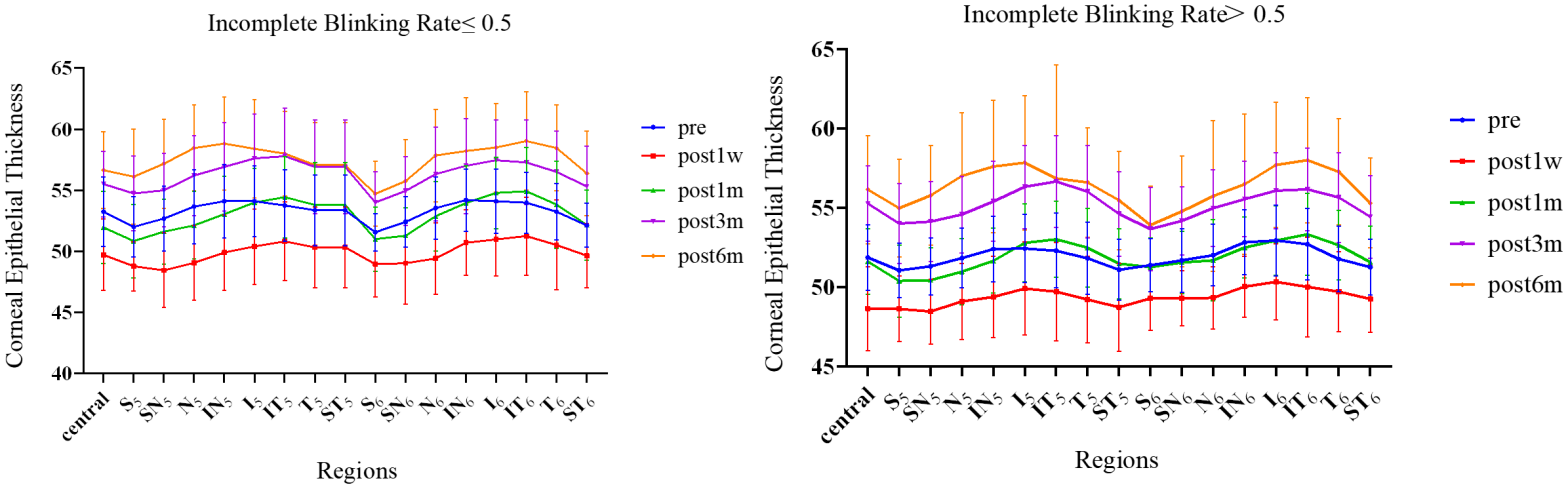
The CET of 17 partitions varies between the groups over time. Error bars represent standard deviations.

Figure 2 shows the changes in CET at different time points after surgery in the two groups of patients. Due to the complete removal of the epithelium during Trans-PRK surgery, CET was significantly lower 1 week postoperatively than preoperatively. One month after surgery, the CET of the IT, T, and ST exceeded the preoperative levels. Although thickness of the other areas had increased compared to at 1 week, this remained lower than the preoperative levels. There was no significant difference in the thickening areas between the two groups. At 3 months, CET in all areas exceeded the preoperative level, and this thickening trend was observed until 6 months. At all postoperative time points, the corneal epithelium thickened more significantly as it approached the peripheral area. Within the 16 zones of the 2–5 mm paracentral area and the 5–6 mm peripheral area, thickening was more significant in N, IN, I, IT, and T than in other regions. Figure 2 shows that S in the peripheral area is significantly thinner than in the paracentral area. We speculated that this was due to eyelid occlusion during the measurement process. There was no significant difference in CET between the groups, and the thickening patterns were consistent.

Table 2 shows the regional differences in CET values between the groups 1 month after surgery. There were significant differences in N, IN, I, IT, and T in the central area (2–5 mm) and peripheral area (5–6 mm). The corneal thickness in these areas in group A was significantly higher than that in group B 1 month after surgery.

**Table 2:** Differences in CET between groups 1 month after surgery (μm, x ± s).

	Zone	Incomplete blinking rate ≤ 0.5	Incomplete blinking rate>0.5	F	*p*	95%CI
	Center	52.25 ± 0.44	51.88 ± 0.29	1.43	0.24	−1.67 to 0.42
2–5 mm						
	S	49.96 ± 0.46	50.39 ± 0.31	0.59	0.45	−1.53 to 0.68
	SN	51.07 ± 0.42	50.48 ± 0.28	1.35	0.25	−0.42 to 1.59
	N	51.79 ± 0.38	50.85 ± 0.26	4.25	0.04	0.33 to 1.84
	IN	52.86 ± 0.43	51.77 ± 0.29	4.42	0.04	0.06 to 2.11
	I	53.75 ± 0.41	52.78 ± 0.28	3.79	0.05	−1.96 to 0.21
	IT	54.11 ± 0.45	53.03 ± 0.30	3.93	0.05	−0.002 to 2.15
	T	53.36 ± 0.43	52.31 ± 0.28	4.24	0.04	0.04 to 2.06
	ST	51.86 ± 0.46	51.52 ± 0.30	0.39	0.54	−0.75 to 1.44
5–6 mm						
	S	50.54 ± 0.41	51.39 ± 0.27	3.04	0.09	−1.83 to 0.12
	SN	50.82 ± 0.41	51.63 ± 0.27	0.49	0.10	−1.78 to 0.17
	N	52.39 ± 0.42	51.33 ± 0.29	4.32	0.04	0.05 to 2.08
	IN	53.68 ± 0.43	52.61 ± 0.29	4.18	0.04	0.03 to 2.10
	I	54.50 ± 0.44	53.11 ± 0.29	6.85	0.01	0.34 to 2.45
	IT	54.79 ± 0.53	53.47 ± 0.35	4.34	0.04	0.06 to 2.57
	T	53.39 ± 0.39	52.45 ± 0.27	4.06	0.05	0.01 to 1.88
	ST	51.46 ± 0.44	51.64 ± 0.27	0.12	0.74	−1.2 to 0.85

### 4.2 Incomplete blinking rate and blinking times

**Figure 3:**
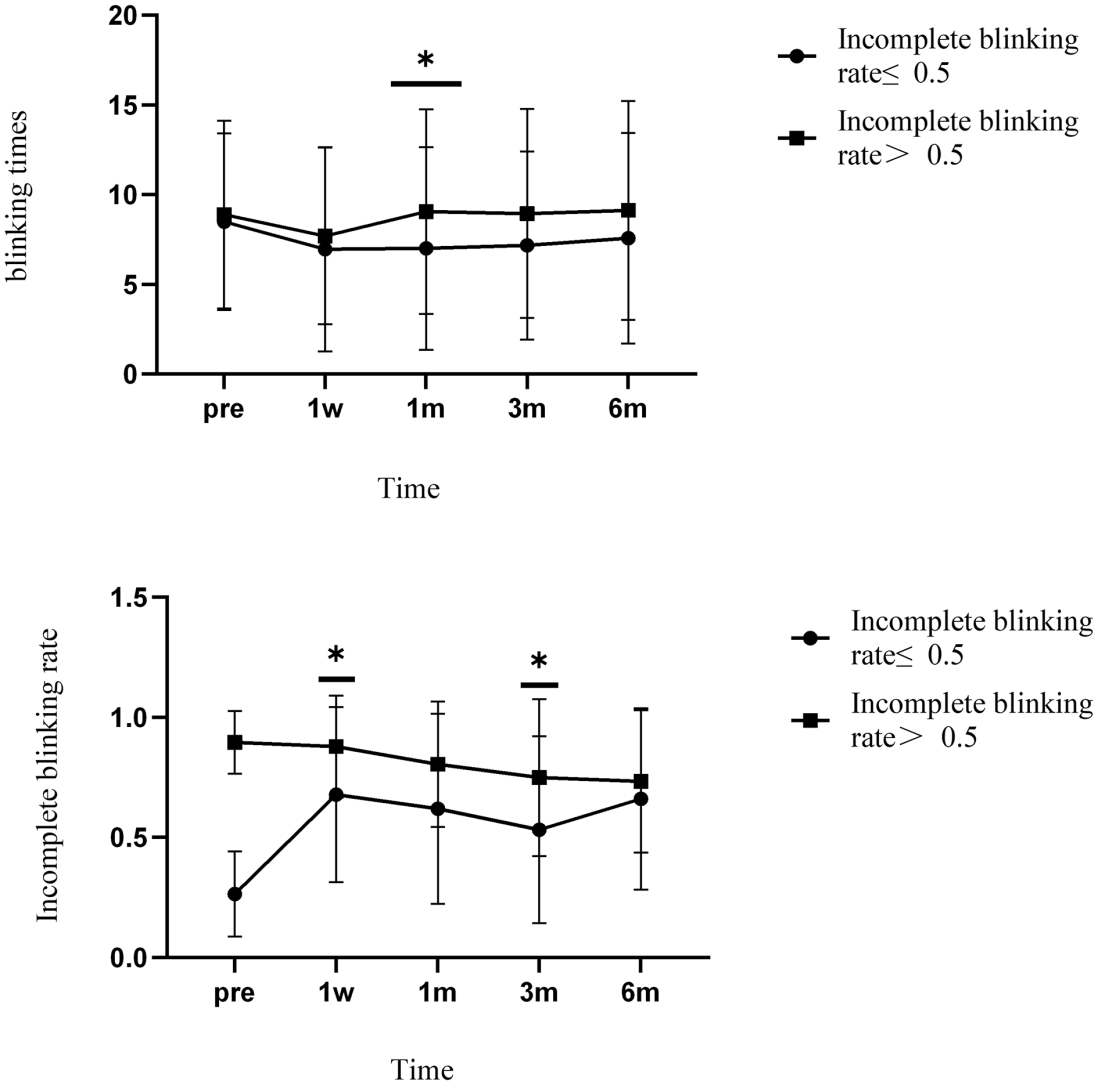
The incomplete blinking rate and blinking times of the two groups varied over time. * Indicates differences between groups. Error bars represent standard deviations.

Patients in group A showed a significant increase in the incomplete blinking rate 1 week after surgery. The rate remained above 0.5 throughout the follow-up period, although it did not return to preoperative levels. The incomplete blinking rate of patients in group B remained above 0.5, at various postoperative time points and showed signs of gradual improvement. There were differences in preoperative rates and postoperative rates at 3 and 6 months (*p* = 0.012, *p* < 0.01). Differences were observed between the two groups at 1 week and 3 months after surgery (*p* = 0.039, *p* = 0.024) (Figure 3).

There was no significant difference in blinking times between the groups over time. Similarly, blink times in both groups decreased 1 week after surgery and stabilized later. There was a significant difference between the groups 1 month after surgery (*p* = 0.017).

### 4.3 Lipid layers thickness

**Figure 4:**
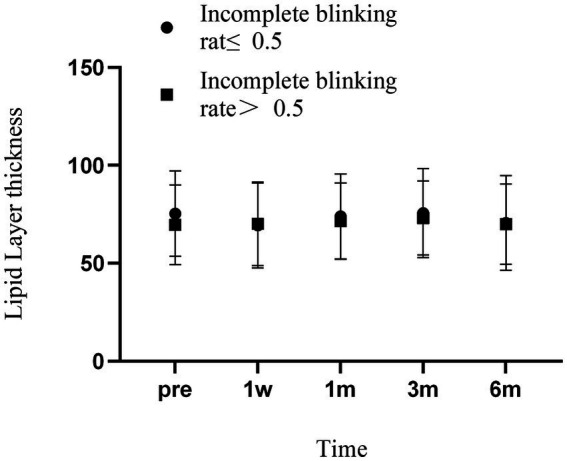
Changes in LLT over time in two groups of patients. Error bars represent standard deviations.

Analysis of the LLT showed that both groups had decreased lipid layer thickness at 1 week, which returned to preoperative levels at 1 month and exceeded preoperative levels at 3 months, followed by small fluctuations. However, the changes in LLT were not significantly different between the groups at any time point two (Figure 4).

### 4.4 Refractive error

**Figure 5:**
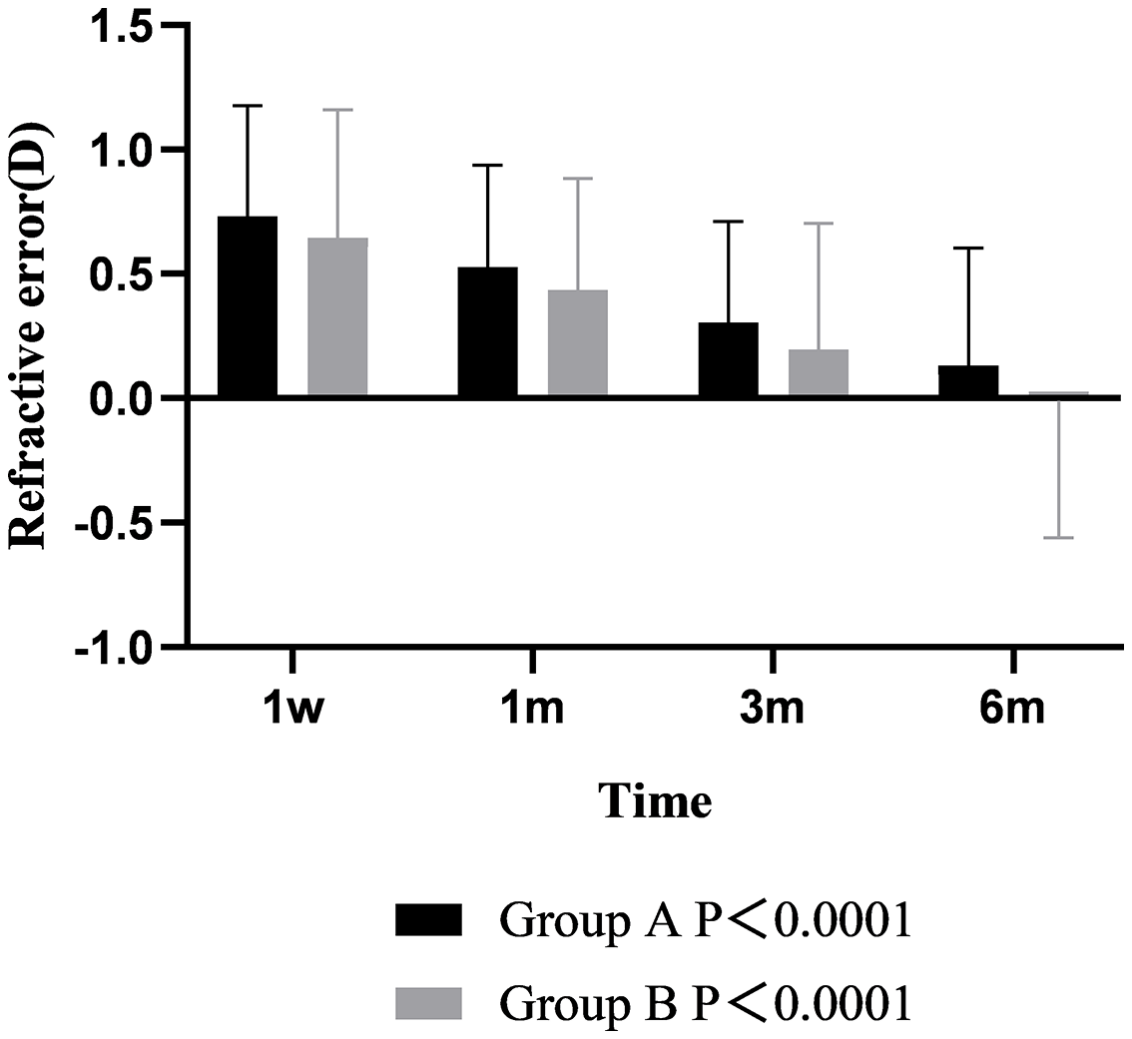
The refractive error of the two groups varied over time.

Postoperative refraction analysis showed that the diopters of the two groups were positive at 1 week after operation, which was slightly overcorrected. The refraction of the two groups gradually decreased from 1 week to 6 months after surgery, and the changes with time were different between the two groups (*p* < 0.001). At 6 months after operation, the refraction of group A was still positive, while the refraction of group B was negative. During the follow-up time, the refraction of group B was always lower than that of group A, but there was no difference between groups (*p* > 0.05) (Figure 5).

### 4.5 Visual acuity

**Figure 6:**
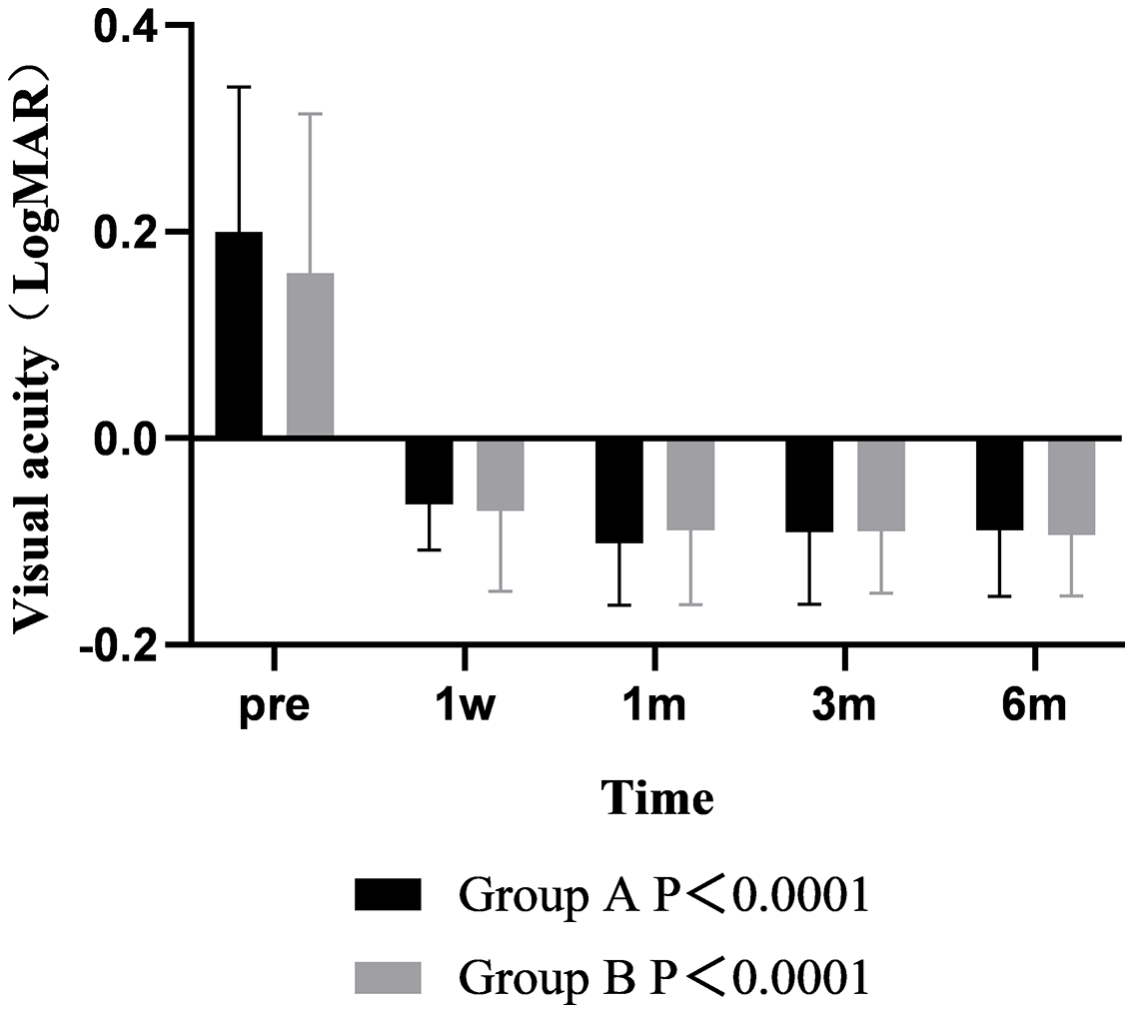
The visual acuity of the two groups varied over time.

The results of visual acuity analysis showed that the postoperative UDVA was better than that before surgery in both groups, and there was significant difference between the two groups over time (*p* < 0.001). The UDVA of the two groups at 1 month after surgery was better than that at 1 week, and the difference was statistically significant (*p* < 0.001). The UDVA remained stable from 1 to 6 months after surgery. There was no significant difference between the two groups at each time point (*p* > 0.05) (Figure 6).

### 4.6 Correlation

**Figure 7:**
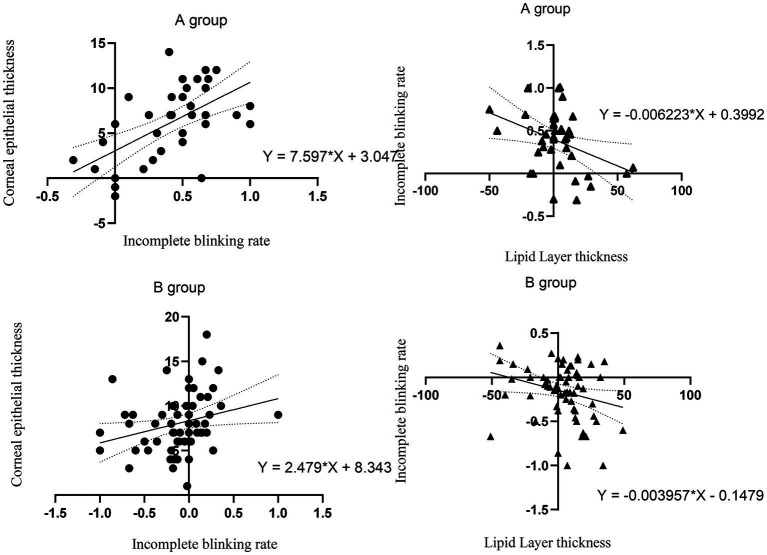
The correlation between incomplete blinking rate and CET. The correlation between LLT and incomplete blinking rate. The thick line represents the regression line. The dashed line represents 95% CI.

We analyzed the correlation between the incomplete blinking rate and changes in CET and found that the higher the incomplete blinking rate after surgery, the more significant the thickening of CET. This indicates that the incomplete blinking rate affects corneal epithelial remodeling to a certain extent. We also found a significant correlation between the incomplete blinking rate and LLT. The higher the incomplete blinking rate, the lower the LLT, which is consistent with previous research (11) (Figure 7).

## 5 Discussion

In this study, we explored the impact of incomplete blinking rate on uneven corneal epithelial remodeling in patients after Trans-PRK. We found that using Trans-PRK to correct myopia led to an increase in uneven epithelial thickening in different corneal regions. In addition, CET was significantly correlated with incomplete blinking rate, and complete blinking rate had a potential impact on LLT.

The decrease in corneal epithelial remodeling and tear film stability after laser ablation is closely related to the loss of corneal nerves (12, 13). Trans-PRK uses an excimer laser to remove the epithelium in one step and ablates the epithelial layer and most of the nerve fibers under the epithelial basement membrane. Trans-PRK is suitable for patients with thin corneas, and when the CET is insufficient to offset the ablation depth, it will ablate a portion of the anterior corneal stroma to some extent. This causes more severe damage to the corneal nerve after Trans-PRK surgery than expected. After corneal nerve injury, the cornea lacks the function of corneal neurotrophins, giving the corneal epithelium a short period of negative or relatively small growth. The gradual growth of corneal nerves leads to corneal remodeling, which is manifested externally by changes in CET (14–17). In our study, CET gradually thickened with the recovery of the corneal nerve. Figure 2 shows that CET showed the greatest increase from 1 to 3 months after surgery, and although it continued to thicken after 6 months, the increase gradually decreased.

When the corneal surface is stimulated, the corneal sensory nerve carries stimulation to the brain through the afferent nerve and then returns through efferent stimulation, reaching the lacrimal gland via the autonomic neural pathway for tear secretion. Therefore, any damage to the ophthalmic branch of the corneal trigeminal nerve or lacrimal gland reflex arc may reduce tear secretion, resulting in a decline in tear film stability (18). After Trans-PRK, the ophthalmic nerve is damaged, corneal sensitivity is reduced, and the afferent neural circuit for tear secretion is damaged. This destroys the functional units of the lacrimal gland on the ocular surface, leading to tear film dysfunction and instability. The decrease in corneal sensitivity does not only abolish the corneal lacrimal gland and blink reflex arc, but also reduces the neurotrophic effect on corneal epithelial cells (3, 19). An abnormal blink reflex pathway can affect the incomplete blinking rate, blinking times, and lead to a decrease in tear secretion and changes in tear quality (20, 21), including decreased lipid secretion and reduced mucin expression (21–24). Patel et al. found that the LLT became thinner after LASIK surgery (25, 26). In our study, the LLT became thinner 1 week after Trans-PRK surgery, and despite a postoperative elevated trend, the LLT remained thinner at 6 months postoperatively than preoperatively. Gao et al. proposed that the LLT recovers to preoperative levels 1 year after surgery (25, 27), which suggests a requirement for an extended follow-up period.

Studies have shown that nerve injury caused by refractive surgery results in dry eye. The decrease in corneal sensitivity, increase in osmotic pressure, and increased frequency of incomplete blinking heightens the inflammatory reaction of the corneal epithelial cells (28). Inflammation caused by postoperative dry eyes can increase the levels of inflammatory cytokines in tears, such as IL − 1a and IL-1b, TNF-a, IL-6, and IL-8, several of which play key roles in epithelial hyperproliferation and keratinization. It has been reported that IL-6 and IL-8 can affect the growth of epithelial cells (29–33). Fabiani et al. found that the corneal epithelium thickened within 7 days after establishing dry eye in a rat model, indicating a significant impact of inflammation on corneal epithelial proliferation (10, 34). Research has shown that Trans-PRK surgery leads to temporary dry eyes, and exposure to a dry environment can significantly increase cell proliferation and central thickness of the corneal epithelium. Dry conditions stimulate cell circulation and proliferation throughout the entire epithelial cell, which is a “stress response” of the epithelium to inflammation (4–6, 9). The changes in epithelial thickness caused by dry eyes have a more profound impact on the peripheral corneal epithelium than on the central region. In our study, we measured corneal thickness in patients after Trans-PRK surgery, and the results were similar to the changes in CET in dry eye patients. Our results show that changes in the rate of incomplete blinking after surgery can, to some extent, affect the amplitude and distribution of corneal epithelial thickening. The higher the rate of incomplete blinking, the thicker the corneal epithelium becomes. Instability of the tear film after refractive surgery is related to inflammation. In this process, an increase in epithelial cell renewal may lead to an increase in CET, and the mechanical friction caused by the increase in blink rate leads to an uneven distribution of CET.

Corneal refractive surgery leads to corneal flattening in the central area and morphological changes, which causes uncoordinated interactions between the ocular surface and the eyelid. After Trans-PRK surgery, the blinking times and incomplete blinking rate both change. In the process of corneal epithelial remodeling, the force exerted by the eyelid on the irregular cornea affects the corneal remodeling process and the distribution of CET.

Trans-PRK with SPT was used in this study. Direct epithelial removal also includes PRK and Trans-PRK. The advantage of SPT is that it can reduce the irregularity of the corneal surface after stromal ablation. However, either type of surgery can cause corneal nerve damage and postoperative dry eye. Therefore, we hypothesized that all three types of surgery could cause corneal epithelial thickening and abnormal blinking, and the incomplete blink rate would affect the distribution of corneal epithelial thickness. The three procedures can be compared in future studies.

The meibomian gland has a known impact on the health of the ocular surface. Atrophy, curvature, or blockage of the meibomian gland will affect the quality and quantity of the lipid layer, which indirectly affects the duration of postoperative dry eyes. In serious cases, this can lead to chronic tear film dysfunction following refractive surgery (22, 35). The lipid layer acts as a lubricant during blinking and the greater the amount of lipid secreted by the meibomian gland, the smaller the mechanical friction of the eyelid on the cornea. Therefore, eyelid force has less impact on corneal remodeling when blinking is normal.

Previous studies have shown that CET after refractive surgery can affect postoperative refractive power, and that postoperative dry eye inflammation can affect the visual quality of patients after surgery (36, 37). This emphasizes that more attention should be paid to corneal epithelial remodeling after refractive surgery, and factors affecting corneal epithelial remodeling should be evaluated from multiple perspectives. Although the degree of meibomian gland atrophy is already included as a criterion for research participants, atrophy increases with age, highlighting the need to evaluate meibomian gland function when considering refractive surgery for older candidates.

Due to the rapid development of electronic products, bad blinking habits have been formed, and the damage to nerves caused by refractive surgery has exacerbated this result. We should pay attention to the patient’s preoperative eye habits and postoperative eye care. The limitation of this article is that, on the one hand, the follow-up time is relatively short, and there is no questionnaire survey conducted on patients’ subjective feelings and eye habits. On the other hand, only Trans-PRK was involved in our study, but previous studies have shown that the corneal epithelium also thickens after FS-LASIK. Unlike Trans-PRK, the corneal epithelium after FS-LASIK thickens more significantly in the central and paracentral regions (38). The FS-LASIK flap and laser ablation result in damage to the subbasal plexus of the corneal epithelium, as well as a decrease in conjunctival goblet cell density during negative pressure aspiration, which aggravates postoperative dry eye (39). Therefore, FS-LASIK may lead to more significant changes in the rate of incomplete blinks as well as changes in lipid thickness. In future research, we will focus on studying the visual quality of patients after surgery and explore whether other surgical methods have the same results.

## 6 Conclusion

Uneven thickening of the corneal epithelia was observed in both groups, and was more pronounced in the peripheral area. In addition, changes in CET after surgery were positively correlated with the incomplete blinking rate in both patient groups. There was a negative correlation between postoperative LLT and incomplete blinking rate.

## Data availability statement

The raw data supporting the conclusions of this article will be made available by the authors, without undue reservation.

## Ethics statement

The studies involving humans were approved by the Ethics Committee of the Eye Hospital of Tianjin Medical University (2022KY-09). The studies were conducted in accordance with the local legislation and institutional requirements. The participants provided their written informed consent to participate in this study. Written informed consent was obtained from the individual(s) for the publication of any potentially identifiable images or data included in this article.

## Author contributions

FY: Conceptualization, Investigation, Methodology, Software, Writing – original draft. HC: Investigation, Writing – original draft. SZ: Supervision, Writing – review & editing. YH: Supervision, Writing – review & editing.
